# Fresh and Cryopreserved Human Umbilical-Cord-Derived Mesenchymal Stromal Cells Attenuate Injury and Enhance Resolution and Repair following Ventilation-Induced Lung Injury

**DOI:** 10.3390/ijms222312842

**Published:** 2021-11-27

**Authors:** Shahd Horie, Hector Gonzalez, Jack Brady, James Devaney, Michael Scully, Daniel O’Toole, John G. Laffey

**Affiliations:** 1 Anaesthesia, School of Medicine, Clinical Sciences Institute, National University of Ireland, H91 TK33 Galway, Ireland; shahd.horie@nuigalway.ie (S.H.); h.gonzalez2@nuigalway.ie (H.G.); J.BRADY8@nuigalway.ie (J.B.); jamesdevaney@gmail.com (J.D.); michael.scully@nuigalway.ie (M.S.); 2 Regenerative Medicine Institute (REMEDI), CÚRAM Centre for Research in Medical Devices, National University of Ireland Galway, Biomedical Sciences Building, H91 TK33 Galway, Ireland; 3 Medicine, School of Medicine, Clinical Sciences Institute, National University of Ireland, H91 TK33 Galway, Ireland; 4 Department of Anaesthesia, Galway University Hospitals, SAOLTA University Health Group, H91 YR71 Galway, Ireland

**Keywords:** acute respiratory distress syndrome, ventilation-induced lung injury, injury, mesenchymal stem/stromal cells, cryopreservation, tissue source

## Abstract

Background: Ventilator-induced lung injury (VILI) frequently worsens acute respiratory distress syndrome (ARDS) severity. Human mesenchymal stem/stromal cells (MSCs) offer considerable therapeutic promise, but the key impediments of clinical translation stem from limitations due to cell source and availability, and concerns regarding the loss of efficacy following cryopreservation. These experiments compared the efficacy of umbilical-cord-derived MSCs (UC-MSCs), a readily available and homogenous tissue source, to the previously more widely utilised bone-marrow-derived MSCs (BM-MSCs). We assessed their capacity to limit inflammation, resolve injury and enhance repair in relevant lung mechanical stretch models, and the impact of cryopreservation on therapeutic efficacy. Methods: In *series 1*, confluent alveolar epithelial layers were subjected to cyclic mechanical stretch (22% equibiaxial strain) and wound injury, and the potential of the secretome from BM- and UC-derived MSCs to attenuate epithelial inflammation and cell death, and enhance wound repair was determined. In *series 2*, anesthetized rats underwent VILI, and later received, in a randomised manner, 1 × 10^7^ MSCs/kg intravenously, that were: (i) fresh BM-MSCs, (ii) fresh UC-MSCs or (iii) cryopreserved UC-MSCs. Control animals received a vehicle (PBS). The extent of the resolution of inflammation and injury, and repair was measured at 24 h. Results: Conditioned medium from BM-MSCs and UC-MSCs comparably decreased stretch-induced pulmonary epithelial inflammation and cell death. BM-MSCs and UC-MSCs comparably enhanced wound resolution. In animals subjected to VILI, both fresh BM-MSCs and UC-MSCs enhanced injury resolution and repair, while cryopreserved UC-MSCs comparably retained their efficacy. Conclusions: Cryopreserved UC-MSCs can reduce stretch-induced inflammation and cell death, enhance wound resolution, and enhance injury resolution and repair following VILI. Cryopreserved UC-MSCs represent a more abundant, cost-efficient, less variable and equally efficacious source of therapeutic MSC product.

## 1. Introduction

Mesenchymal stem/stromal cells (MSCs) show promise as a therapeutic strategy for sepsis and acute respiratory distress syndrome (ARDS). MSCs were observed to modulate the inflammatory process, improve alveolar epithelial barrier function, attenuate lung injury and reduce the overall mortality in diverse preclinical sepsis and ARDS animal models [1,2,3,4,5,6,7], including our model of *E. coli*-induced pneumonia [8,9] and repair from ventilator-induced lung injury (VILI) [10]. VILI is an inflammatory process that can result from certain mechanical ventilation strategies, which are necessary to ensure adequate oxygenation but often worsen ARDS. The mechanisms of action of MSCs in VILI are numerous, including the secretion of a range of paracrine mediators and microparticles that can ameliorate the evolution of injury and inflammation, as well as promote tissue repair and recovery [11]. Importantly, MSCs demonstrate utility in human lungs ex vivo [12], and MSCs are well tolerated, showing promising efficacy in a number of phase I/phase II clinical trials [13].

As early phase trials progress, three key issues in relation to the clinical translation of MSCs have been highlighted which are the source of the cell, the protocol of cell expansion, and the mechanism of storage and transport. The industrialization of bone marrow (BM) MSC production for large-scale phase III clinical trials necessitates extensive culture expansion from each bone marrow donation in order to secure suitable doses for systemic delivery [14]. MSCs, which are expanded in cultures, exhibit telomere shortening, which is one factor causative of senescence and an eventual form of cell exhaustion and cessation of proliferation. [15,16]. Other expansion protocol factors can also contribute to phenotypic changes in the cell therapy which could diminish efficacy, reduce cell longevity after engraftment and hamper their regenerative and immunomodulatory properties [16].

The human umbilical cord is a rich source of MSCs and preparing them from this tissue has many obvious advantages when compared to the traditional alternatives. These advantages are particularly clear with regard to the production volumes of the MSCs without introducing senescence. The umbilical cord is also easily procured, as it is a waste tissue where harvesting does not require any sort of invasive procedure as with bone marrow or adipose MSCs. From one donor, the umbilical cord can produce more early-passage MSCs (by one order of magnitude) than a typical bone marrow donation, instead facilitating the manufacturing of large quantities of the lower-passage MSCs to provide the greatest immunomodulatory capacity [17]. Due to the age of their source, all UC-MSCs are essentially the same age, which increases the homogeneity of the final cell product, enhancing reproducibility in the recipient [18].

Finally, the majority of previous studies using MSCs use them as harvested directly from culture, but this approach may only be of utility for chronic diseases and even then is of reduced practicality. It remains to be conclusively proven whether efficacy is retained when MSCs are administered immediately following cryopreservation; therefore, this is a critical step on the translational path. Differences in MSC characteristics, particularly their immune modulation capabilities, were linked to their source of origin and cryopreservation status and, as such, direct comparison studies are required [19,20,21,22].

We hypothesised that UC-MSCs would modulate inflammation and enhance repair after VILI, in a comparable manner to the “gold standard” BM-MSCs, and that cryopreservation would not alter MSC efficacy.

## 2. Results

### 2.1. Cyclic Stretch-Induced Epithelial Injury In Vitro

**Figure 1:**
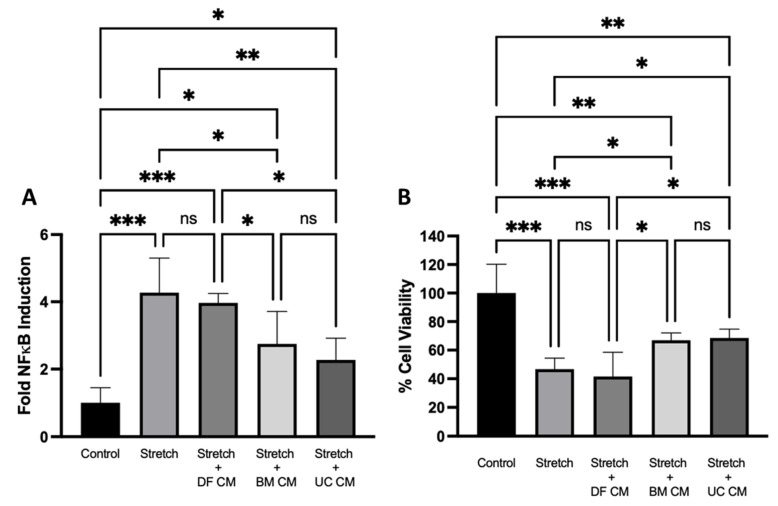
**UC-MSC CM attenuates stretch injury in vitro**. Stretch-induced NFκB induction in alveolar epithelial cells was significantly reduced with BM-MSC CM and UC-MSC CM (**A**). The stretch-induced decrease in cell viability was also ameliorated with BM-MSC CM and UC-MSC CM treatment (**B**). Note: *, **, *** = *p* < 0.05, 0.01, 0.001, respectively. ns = not significant. *n* = 3–5 per group. Control = Non-stretched cells in MEM-α medium. Stretch = stretched cells in MEM-α medium. Stretch + DF CM = stretched cells in Dermal Fibroblast CM. Stretch + BM CM = stretched cells in BM-MSC CM. Stretch + UC CM = stretched cells in UC-MSC CM.

**Resolution of In Vitro Stretch Injury**: 120 h of mechanical cyclic stretch caused a significant induction of the NFκB transcription factor (*p* < 0.001) and a significant decrease in the viability of alveolar epithelial cells (*p* < 0.001) (Figure 1). BM-MSC conditioned medium (CM) significantly attenuated NFκB activation (*p* < 0.05), as did UC-MSC CM (*p* < 0.01) (Figure 1A). Both BM-MSC CM and UC-MSC CM significantly reversed the decrease in cell viability (*p* < 0.05) in comparison to the DF CM control (Figure 1B).

### 2.2. Pulmonary Epithelial Wound Healing In Vitro

**Figure 2:**
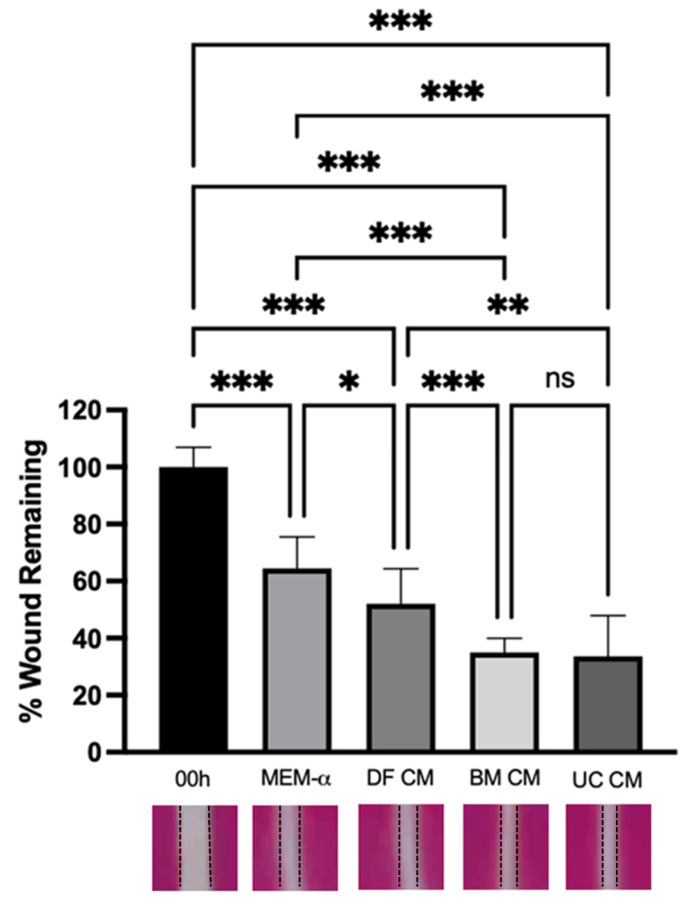
**UC-MSC CM accelerates repair following wound injury in vitro.** BM-MSC CM and UC-MSC CM significantly enhanced wound closure in vitro, in comparison to MEM-α and DF CM controls. Note: *, **, *** = *p* < 0.05, 0.01, 0.001, respectively. ns = not significant. *n* = 3–5 per group. 00h = Time 0 h, at which scratch wounds were implemented. MEM-α = scratched layers in MEM-α medium at 48 h. DF CM = scratched layers in DF CM at 48 h. BM CM = scratched layers in BM-MSC CM at 48 h. UC CM = scratched layers in UC-MSC CM at 48 h.

**Wound Healing:** Treatment with BM-MSC CM or UC-MSC CM significantly enhanced wound restitution in alveolar epithelial cells following scratch wounding in vitro, when compared to the MEM-α control (*p* < 0.001) and DF CM control (*p* < 0.01 and 0.05, respectively) (Figure 2).

### 2.3. Injury Resolution Following In Vivo Ventilation-Induced ARDS

**Figure 3:**
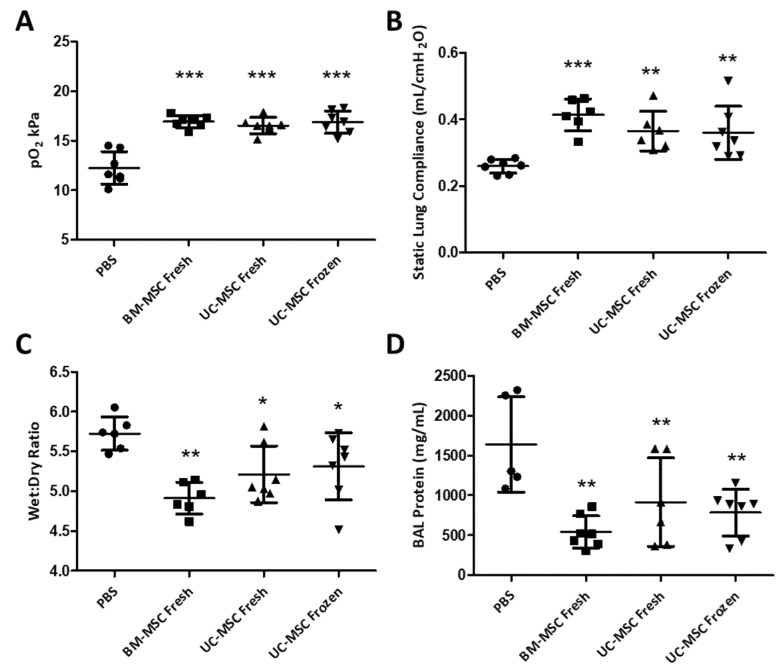
**Cryopreserved UC-MSCs restore lung function and the permeability barrier post VILI.** BM-MSCs and UC-MSCs comparably restored arterial oxygen (**A**) as did thawed cryopreserved UC-MSCs (**A**). The decrease in static lung compliance was also restored by both cell sources (**B**) while thawed cryopreserved UC-MSCs retained their efficacy (**B**). Alveolar fluid clearance as depicted by lung wet:dry ratio and BAL protein concentrations was significantly restored by both BM-MSCs and UC-MSCs (**C**,**D**), while thawed, cryopreserved UC-MSCs were comparably efficacious (**C**,**D**). Note: *, **, *** = *p* < 0.05, 0.01, 0.001, respectively versus PBS control groups. *n* = 7 animals per group.

**Recovery of Lung Function:** VILI caused a significant decrement in oxygenation, lung compliance and lung permeability compared to protective ventilation (Figure 3). Both fresh BM-MSCs and UC-MSCs restored arterial oxygenation (*p* < 0.001) (Figure 3A) when compared to the vehicle (PBS) control group. Importantly, thawed, cryopreserved UC-MSCs also restored arterial oxygenation (*p* < 0.001), demonstrating that these cells retained efficacy post cryopreservation. The decrement in static lung compliance induced by VILI was restored by BM-MSCs and UC-MSCs (*p* < 0.001 and 0.01, respectively), while thawed, cryopreserved UC-MSCs were similarly effective (*p* < 0.01) (Figure 3B). BM-MSCs and UC-MSCs increased the restoration of alveolar barrier permeability, as shown by a reduction in the lung wet:dry weight ratio (*p* < 0.01 and 0.05, respectively) (Figure 3C) and a reduction in the accumulated total protein in the airspace (*p* < 0.01) (Figure 3D). The thawed cryopreserved UC-MSCs retained their efficacy and also restored alveolar fluid clearance (*p* < 0.05) and protein concentrations (*p* < 0.01) to normal levels.

**Figure 4:**
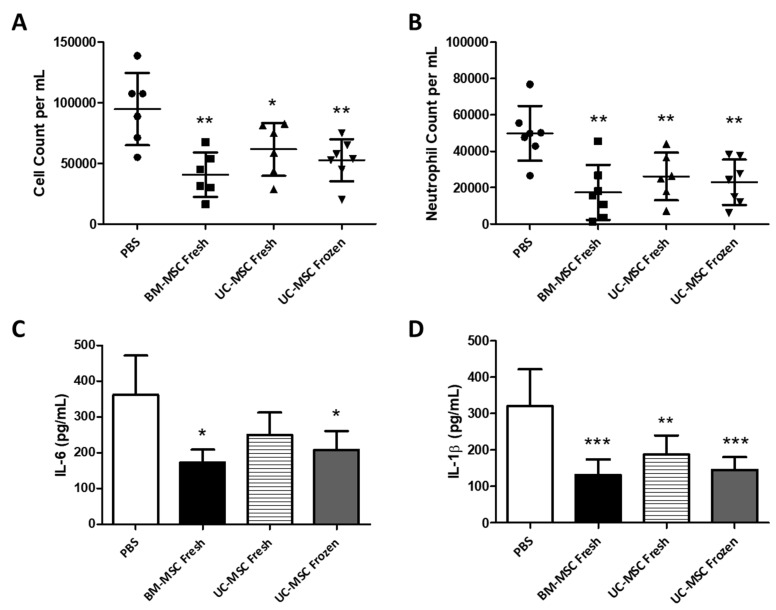
**Cryopreserved UC-MSCs modulate inflammatory cell influx and cytokine release.** All cell treatment groups significantly resolved cell and neutrophil infiltration into the lung (**A**,**B**) while cryopreserved UC-MSC treatment was comparable in efficacy (**A**,**B**). Fresh BM-MSCs and thawed, cryopreserved UC-MSCs significantly attenuated BAL IL-6 (**C**) when compared to PBS control. All cell treatments ameliorated BAL IL-1β levels (**D**). Note: *, **, *** = *p* < 0.05, 0.01, 0.001, respectively, versus PBS control groups. *n* = 7 animals per group.

**Modulation of the Inflammatory Response**: VILI caused a significant inflammatory response (Figure 4). Fresh BM-MSCs and both fresh and cryopreserved UC-MSCs comparably decreased alveolar cell counts (*p* < 0.01, 0.05 and 0.01, respectively) (Figure 4A) and alveolar neutrophil counts (*p* < 0.01) (Figure 4B) when compared to the vehicle (PBS) control group. IL-6 release, induced following VILI, was significantly attenuated by fresh BM-MSCs (*p* < 0.05) (Figure 4C). While IL-6 concentrations were lower in the BAL of animals that received freshly delivered UC-MSCs, this was not statistically significant (Figure 4C). In contrast, thawed, cryopreserved UC-MSCs reduced alveolar IL-6 concentrations (*p* < 0.05 (Figure 4C). All MSC treatment groups demonstrated significantly reduced alveolar IL-1β concentrations (*p* < 0.001, 0.01 and 0.001, respectively) when compared to the control group (Figure 4D).

**Figure 5:**
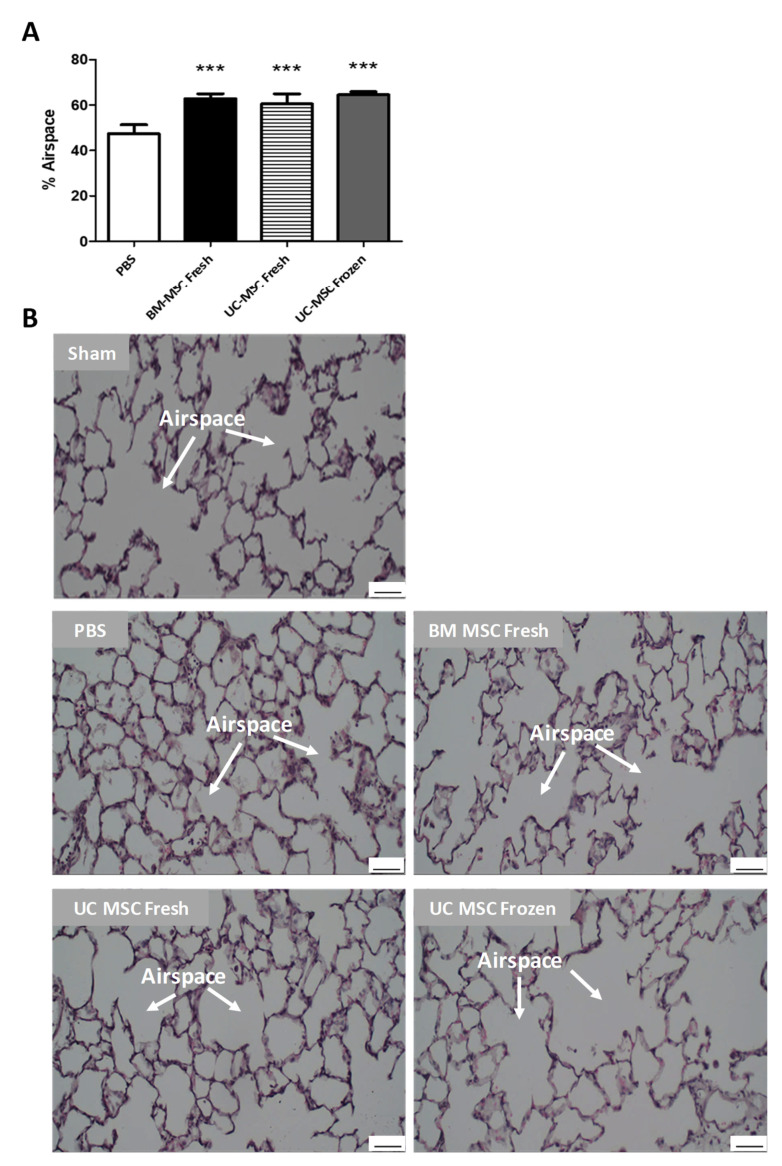
**Cryopreserved UC-MSCs restore lung histologic structure.** Fresh BM-MSCs and fresh UC-MSCs comparably restored alveolar airspace when compared to PBS control (**A**). Thawed, cryopreserved UC-MSCs restored lung structure with similar efficacy (**A**). Representative images of alveolar structure and airspace are depicted above (**B**). Note: *** = *p* < 0.001 versus PBS control. *n* = 4 animals per group. Sham = healthy uninjured control. PBS = animals that received PBS. BM MSC Fresh and UC MSC Fresh = animals that received freshly cultured BM-MSCs or UC-MSCs, respectively. UC MSC Frozen = animals that received cryopreserved UC-MSCs. Scale bar is 50 microns.

**Restoration of Lung Structure:** VILI caused significant alveolar epithelial structural damage. Treatment with BM-MSCs and UC-MSCs, whether fresh or following cryopreservation, significantly enhanced the restoration of lung histologic structure post VILI (*p* < 0.001), depicted as percentage airspace (Figure 5A) and the amelioration of interstitial and alveolar inflammatory cell infiltration (Figure 5B).

## 3. Discussion

This study demonstrates several novel findings and concludes that UC-MSCs are comparably effective to the “gold standard” BM-MSCs for enhancing repair in the injured epithelium and lung and that UC-MSCs retain efficacy when delivered thawed, post cryopreservation. These findings have important implications for the translation and production of MSC therapies.

### 3.1. The Secretome of UC-MCS and BM-MSCs Comparably Rescues the Injured Lung Epithelium

Treatment with the secretome of either BM-MSCs or UC-MSCs comparably attenuated mechanical stretch injury and enhanced repair following wound injury in vitro. Similar findings were observed in previous studies [8,10] and this further supports the hypothesis that the mechanism of therapeutic action of MSCs involves, in part, the release of paracrine mediators.

### 3.2. UC-MSCs and BM-MSCs Comparably Restore Lung Function

This study demonstrated for the first time, that UC-MSCs are comparably efficacious to BM-MSCs, in restoring oxygenation and compliance in a rat model of recovery following VILI. We previously reported this finding for fresh BM-MSCs in VILI [23], but this is the first study to directly compare the efficacy of BM-MSCs to UC-MSCs in this preclinical model. This study also observed that UC-MSCs retained their efficacy to achieve the aforementioned effects after cryopreservation, which is a significant advance for clinical translation in terms of production and delivery. Previous studies reported that cryopreserved BM-MSCs were efficacious in restoring oxygenation and compliance in a rat model of pneumonia [8] and this can be considered alongside the findings of this study to further support the use of cryopreserved MSCs.

Administration of either BM-MSCs or UC-MSC cells was also comparable in the restoration of alveolar membrane integrity, as both treatments were shown to equally restore alveolar fluid clearance and reduce protein concentration in the injured lung. The administration of BM-MSCs was previously shown to lower protein concentrations and fluid retention in the lungs of animals with VILI, but this study is the first to obverse these findings for UC-MSCs in VILI. Furthermore, cryopreservation did not hinder the efficacy of UC-MSCs to restore alveolar membrane integrity following VILI, which is a novel finding in this pre-clinical model of ARDS. One study previously observed that cryopreserved UC-MSCs significantly attenuated protein concentration in the lungs of rats with pneumonia [24]. Overall, these studies further support the use of cryopreserved MSCs in future clinical studies.

### 3.3. UC-MSCs and BM-MSCs Comparably Modulate the Inflammatory Response

The delivery of fresh, either BM-MSC or UC-MSC cell doses, significantly modified the inflammatory response to VILI as evidenced by resolved inflammatory cell infiltration in the injured lung, and cryopreservation did not hamper the efficacy of this MSC response. Similar observations were reported in rat models of *E.coli*-induced lung injury [8,9], but this is the first study to observe these findings in a VILI model.

The administration of MSCs in this animal model revealed a modified BAL cytokine profile. Freshly delivered BM-MSCs significantly relieved the release of IL-6, whereas UC-MSCs did not. Interestingly, the cryopreserved cells did show a significant benefit. MSCs that are harvested on the same day, pooled, cryopreserved and then thawed for delivery, are likely to present as a more homogenous cell dose. Cryopreservation can therefore reduce the variability in MSC efficacy that can be attributed to either cell culture conditions or donor differences. BM-MSCs and UC-MSCs were previously observed to reduce BAL IL-6 in a rat pneumonia model [9]. Finally, fresh BM-MSCs, and either fresh or cryopreserved UC-MSCs, significantly reduced pro-inflammatory IL-1β release in the BAL. Overall, it is clear that neither the cell source nor cryopreservation can hinder the immunomodulatory effects of MSCs in this VILI animal model, and this agrees with previously published reports in other models of ARDS [8,9].

### 3.4. UC-MSCs and BM-MSCs Comparably Restore Lung Structure

Alveolar airspace and lung structure was significantly restored by MSC treatment, confirming that the MSCs from either BM or UC sources are equally efficacious, even after cryopreservation, in promoting resolution from injury in VILI. Similar observations in other preclinical models of ARDS were reported [8,24].

## 4. Materials and Methods

### 4.1. Cell Culture

**A549/NF-κB-luciferase Cell Line Culture:** NFκB inflammatory signaling is strongly involved in stretch-induced lung inflammation and injury, as well as ARDS development [25,26,27]. A549/NFκB-luciferase cells (Panomics, Fremont, CA, USA) were purchased as cryopreserved 3-passage culture and used at passages 4–10. These cells have an integrated chromosomal luciferase reporter construct that is regulated by NFκB, and are used for examining NFκB transcription factor activity in vitro. Breifly, A549 cells were co-transfected with a NFκB luciferase reporter plasmid and hygromycin-resistant plasmid, and then selected with hygromycin in culture. A TNF-α and luciferase assay was then used to select hygromycin-resistant cell clones. A549/NFκB-luciferase cells were passaged in RPMI-1640 growth medium (Sigma-Aldrich Ireland Ltd., Wicklow, Ireland) supplemented with 10% fetal bovine serum (FBS) (Sigma-Aldrich), 1% penicillin G (100 U/mL) and streptomycin (100µg/mL) solution (Sigma-Aldrich), 1% L-glutamine (0.2 mg/mL) (Sigma-Aldrich) and hygromycin (50 μg/mL final) (Roche Life Science, Penzberg, Germany). These cells were maintained in a humidified (95%) tissue culture incubator saturated with a gas mixture containing 5% CO_2_ and 20% O_2_ in air at 37 °C. These cells were sub-cultured with 0.025% trypsin/0.05 mM EDTA (GIBCO^®^, Invitrogen Corporation, Grand Island, NY, USA) and cryopreserved in CryoStor™ cell preservation medium (Sigma-Aldrich).

**MSC and Dermal Fibroblast (DF) Isolation, Culture and Expansion:** Human BM-MSCs and UC-MSCs were isolated as previously described [9,28] and used at passages 1–3 for all experiments. Briefly, for BM-MSCs, bone marrow aspirates were obtained from healthy donors after ethical consent and were subjected to Ficoll density gradient centrifugation (GE Healthcare, Chalfont St. Giles, UK). Mononuclear cells were selected by plastic adherence and cell surface marker expression. For UC-MSCs, umbilical cord tissue was ethically obtained after consent and physically disassociated. The tissue was then subjected to enzymatic breakdown in culture media containing Collagenase 1 (2 mg/mL) (Sigma Aldrich) at 37 °C for less than one hour. The cells were filtered and a single cell suspension was obtained. Cells were selected by plastic adherence and surface marker expression. MSCs were cultured in Alpha Minimum Essential Eagle Medium plus Glutamax (MEM-α) (GIBCO^®^) supplemented with 10% FCS, penicillin G (100 U/mL) streptomycin (100 μg/mL) and FGF-1 (10 ng/mL) (PeproTech EC Ltd., London, UK). Cells were maintained in 95% humidity, 5% CO_2_ and 2% O_2_ (hypoxia) at 37 °C. These cells were sub-cultured with 0.025% trypsin-0.05/mM EDTA and cryopreserved in CryoStor™ cell preservation medium (200 μL per 1 million cells). DFs were used as control cells. DFs were derived from skin punch biopsies (3 mm), secured and cultured in 6-well plates (Sarstedt, Newton, NC, USA) until 80–90% confluent, then expanded and maintained as described above. For animal dosing, cells were reconstituted in 1 mL of phosphate-buffered saline (PBS) (Sigma-Aldrich). Cryopreserved MSCs were stored for up to two months and cell viability after thawing was between 95–97% as determined by trypan blue exclusion.

**Preparation of CM from DFs and MSCs:** On day 1, the cells (passage 1–3) were seeded in a T175 tissue culture flask (Sarstedt) at 1 × 10^5^ cells/cm^2^ in complete MEM-α medium (20 mLs). Forty-eight hours later (Day 3), the cells were washed three times in PBS solution before the addition of 20 mLs of serum-free MEM-α medium. The CM was collected 48 h later (Day 5) and then stored at −80 °C for later use. The CM was discarded after two freeze–thaw cycles.

### 4.2. Cyclic Mechanical Cell Stretch

As previously described [25], A549/NFκB-luciferase cells were seeded to laminin-coated 6-well Bioflex plates (Flexcell International, Burlington, NC, USA), mounted onto the Flexcell FX-4000T^®^ Tension Plus^®^ baseplate (Flexcell International) and subjected to 22% equibiaxial stretch at a frequency of 0.1 Hz for 120 h. Non-stretched cells were used as control cells. Cells were maintained in their respective treatments for the entire 120 h. Following stretch, the cells were harvested for luciferase and viability assays.

### 4.3. Stretch Injury Assessment

**Luciferase Assay for NFκB Activity:** Cells were harvested and pelleted then mixed with 50 μL of SolarGlow SuperBright (Molecutools, Dublin, Ireland) luciferase assay substrate for 5 min. Luminescence was measured in a VICTOR™ X plate reader (Perkin Elmer, Boston, MA, USA).

**Viability Assay:** Metabolic/mitochondrial activity was assessed by the thiazolyl blue tetrazolium bromide (MTT) (Sigma-Aldrich) assay, as previously described [29]. Briefly, 5% of intact harvested cells were incubated with 100 μL of MTT solution (100 μg/mL final concentration) in complete RPMI medium, in a tissue culture incubator (5% CO_2_) for 2 h. 50 μL of DMSO was then added to each sample, and samples were kept at room temperature on an orbital mixer for 30 min. Absorbance values were measured at 550 nm.

### 4.4. In Vitro Scratch Wounds

A549/NFκB-luciferase cells were seeded at 1 × 10^5^ cells per cm^2^ in a 24-well plate (Sarstedt) and left to reach confluence for 48 h. Single scratch wounds were generated with a 1 mL pipette tip (Sarstedt). The cells were washed with PBS and their respective treatments were added. Wound restitution was assessed over 48 h in scanned images using the edge finding function in image analysis software (GNU Image Manipulation Program).

### 4.5. Rodent Model of Resolution Post Ventilation-Induced Lung Injury

All work was approved by the Animal Care in Research Ethics Committee of the National University of Ireland Galway and conducted under license from the Health Products Regulatory Agency Ireland (License B100/4253). Specific-pathogen-free adult male Sprague Dawley rats (Charles River Laboratories, Kent, UK), weighing between 350–450 g, were used in all experiments.

**Induction of VILI:** As previously described [10], rats were anaesthetized with isoflurane gas and intravenous access was obtained via the tail vein. A laryngoscopy was performed and a 14 G catheter (BD Insyte^®^, Becton Dickinson Ltd., Oxfordshire, UK) was used to intubate the animal [30] for ventilation using a small animal ventilator (CWE SAR 830 AP; CWE Inc., Ardmore, PA, USA). Anaesthesia was maintained with repeated boli of Alfaxan^®^ (Alfaxadone 0.9% (*w*/*v*) and alfadolone acetate 0.3% (*w*/*v*); Vétoquinol SA, Lure Cedex, France) and paralysis with cisatracurium besylate 0.5 mg·kg^−1^ (GlaxoSmithKline, Dublin, Ireland). Following baseline ventilation, static compliance was measured and VILI was induced using the following ventilator settings: Fi_O2_ of 0.3, P_insp_ 35 cmH_2_O, respiratory rate 18 min^−1^, and PEEP 0 cmH_2_O. Following development of severe VILI, as evidenced by a 50% decrease in respiratory static compliance, injurious ventilation was discontinued, and the animals were allowed to recover [8].

**Experimental Design:** Fifteen minutes post induction of VILI, animals were randomized to receive intravenous administrations of 1 × 10^7^ MSCs/kg that were: (i) fresh BM-MSCs; (ii) fresh UC-MSCs; or (iii) thawed, cryopreserved UC-MSCs. Control animals received PBS solution and the extent of inflammation and injury resolution in all groups was measured at 24 h.

### 4.6. Assessment of Lung Injury and Recovery

**In Vivo Assessment:** At 24 h post induction of VILI, animals were re-anaesthetized as described above, intravenous access was obtained via tail vein, and a tracheostomy tube was inserted [10]. Following gaining intra-arterial access, anaesthesia was maintained with Alfaxan^®^and paralysis with cisatracurium besylate, and mechanical ventilation commenced. Arterial blood pressure, airway pressure, lung static compliance and arterial blood gas analyses were performed as previously described [8].

**Ex Vivo Analyses:** Following exsanguination under anesthesia, bronchoalveolar lavage (BAL) was performed, and BAL fluid differential leukocyte counts were completed. BAL concentrations of IL-6 and IL-1β, were determined using ELISA (R&D Systems, Oxfordshire, UK) and BAL protein concentrations were also measured (Micro BCA; Pierce, IL, USA) as per the manufacturers guidelines. The left lung was isolated and fixed in 4% paraformaldehyde solution (PFA), sectioned, stained with haematoxylin/eosin and histologic lung damage determined using quantitative stereological techniques [31]. All ex vivo analyses were performed by blinded investigators.

### 4.7. Statistical Analysis

The distribution of the data was tested for normality using Kolmogorov–Smirnov tests. Data sets were analysed by a one-way analysis of variance (ANOVA), with a post hoc Student–Newman–Keuls test, for between group comparisons. Data are presented as mean ± standard deviation (SD). A *p* value of <0.05 was considered statistically significant. The power and sample size of the in vivo study was determined using estimations of variance for 2 key indices of ARDS (oxygenation and compliance) based on previously published data [10,32] and the G*Power 3 program [33]. The study included *n* = 7 animals per group and the power of the study was >0.80.

## 5. Conclusions

The study confirms that cryopreservation has no deleterious effect on the therapeutic efficacy of UC-MSCs in this injury and repair model, and this further supports the use of MSCs for different clinical presentations of ARDS. UC-MSCs represent a more readily available and cost-effective source of MSCs that is suitable for large-scale expansion and industrial production. The integrity and consistency of this cell product can be further maintained with cryopreservation without the loss of therapeutic efficacy, and signifies a huge advantage for the use of MSCs as a therapy for patients with ARDS.

## Data Availability

Data is available from the authors upon reasonable request.

## Abbreviations

ARDS	Acute Respiratory Distress Syndrome
MEM-α	Alpha Minimum Essential Eagle Medium
ANOVA	Analysis of Variance
BM	Bone Marrow
BAL	Bronchoalveolar Lavage Fluid
CM	Conditioned Medium
CINC-1	Cytokine-Induced Neutrophil Chemoattractant 1
DF	Dermal Fibroblast
ELISA	Enzyme Linked Immunosorbent Assay
EDTA	Ethylenediamine Tetra Acetic Acid
FGF	Fibroblast Growth Factor
IFN-γ	Interferon Gamma
IL-1β	Interleukin-1 Beta
IL-6	Interleukin-6
KGF	Keratinocyte Growth Factor
MSC	Mesenchymal Stem/Stromal Cell
NFκB	Nuclear Factor Kappa B
PaO_2_	Partial pressure of arterial oxygen
PBS	Phosphate-Buffered Saline
PEEP	Positive End-Expiratory Pressure
SD	Standard Deviation
MTT	Thiazolyl Blue Tetrazolium Bromide
TNF-α	Tumour Necrosis Factor-Alpha
UC	Umbilical Cord
VILI	Ventilator-Induced Lung Injury
